# Extra Virgin Olive Oil and Cardiovascular Protection in Chronic Kidney Disease

**DOI:** 10.3390/nu14204265

**Published:** 2022-10-12

**Authors:** Giulia Marrone, Silvia Urciuoli, Manuela Di Lauro, Jessica Ruzzolini, Francesca Ieri, Pamela Vignolini, Francesca Di Daniele, Cristina Guerriero, Chiara Nediani, Nicola Di Daniele, Annalisa Noce

**Affiliations:** 1 UOC of Internal Medicine—Center of Hypertension and Nephrology Unit, Department of Systems Medicine, University of Rome Tor Vergata, 00133 Rome, Italy; 2 PHYTOLAB (Pharmaceutical, Cosmetic, Food Supplement, Technology and Analysis)—DiSIA, University of Florence, 50019 Florence, Italy; 3 Department of Experimental and Clinical Biomedical Sciences “Mario Serio”, University of Florence, 50121 Florence, Italy; 4 School of Applied Medical, Surgical Sciences, University of Rome Tor Vergata, 00133 Rome, Italy; 5 Department of Systems Medicine, UOSD of Dermatology, University of Rome Tor Vergata, 00133 Rome, Italy

**Keywords:** cardiovascular protection, extra virgin olive oil, chronic kidney disease, inflammatory state, oxidative stress, minor phenolic compounds, atherogenic indices, carotid intima-media thickness

## Abstract

The high mortality related to chronic kidney disease (CKD) is not only due to the disease itself; in fact, CKD also represents an important risk factor for cardiovascular (CV) morbidity and mortality. Among the functional foods that seems to have cardioprotective action, extra virgin olive oil (EVOO) plays a pivotal health-promoting role. The aim of this study was to evaluate the possible cardioprotective effects of an EVOO containing a very high content (>900 ppm) of minor phenolic compounds (MPCs). The selected EVOO was analyzed by HPLC-DAD-MS to establish the MPC content. The Olea extract obtained from the selected EVOO was tested against the RAW 264.7 cell line in order to investigate its anti-inflammatory activity. We enrolled 40 CKD patients under conservative therapy for in vivo clinical testing. All CKD patients consumed 40 mL/day of raw EVOO for 9 weeks (T1). At baseline (T0) and at T1, we monitored the patients’ blood and urinary parameters. The patients’ body composition was assessed using bioelectrical impedance analysis and the carotid intima-media thickness (CIMT) using ultrasound imaging. At T1, we observed a decrease in inflammatory parameters, CIMT, and oxidative stress biomarkers. We also noticed improvements in lipid and purine metabolism, atherogenic indices, and body composition. Thus, this study highlighted the cardioprotective action of EVOO in nephropathic patients.

## 1. Introduction

The incidence of chronic kidney disease (CKD) has increased significantly in the last three decades due to the explosive spread of related risk factors such as arterial hypertension, diabetes mellitus, metabolic syndrome, etc. In 2016, CKD reached 13th place in the leading causes of death in the world, and it has been hypothesized that by 2040, it will be the 5th leading cause of death in the world [1]. The high mortality related to CKD cannot be explained only by the disease itself; rather, CKD also represents an important risk factor for cardiovascular (CV) mortality and morbidity [2,3]. In fact, the presence of CKD has been observed to be associate with the onset of a series of comorbidities that are risk factors for CV diseases. Among these comorbidities, special mention is due to alterations of calcium–phosphorus metabolism [4,5], hyperhomocysteinemia [6,7], the increase of asymmetric dimethylarginine (ADMA), malnutrition, uremic sarcopenia [8,9], the accumulation of toxic substances, including those derived from the gut microbiota [10,11], a low-grade chronic inflammatory state, and dyslipidaemia and metabolic acidosis [12,13,14,15]. All these factors contribute to the development of accelerated atherosclerosis in which the primum movens is represented by endothelial dysfunction (ED) [16,17]. Physiologically, the endothelium can be considered a “pervasive” organ that performs numerous functions, among which is the maintenance of vascular tone. In fact, it regulates vascular permeability, the immune response, and angiogenesis [17]. During CKD, ED is amplified and all the previously mentioned CV risk factors contribute to its genesis. As a matter of fact, an increase in ED prevalence was previously observed in relation to CKD stage [18]. Moreover, several studies have demonstrated a worsening of ED directly related to the reduction of the glomerular filtration rate (GFR) in CKD patients [17]. Low-grade chronic inflammatory states are due to an increased production of pro-inflammatory cytokines (interleukin-IL-6, tumor necrosis factor-TNF-α, IL-1β) in association with an enhanced formation of reactive oxygen species (ROS), with consequent induction of oxidative stress (OS). Both inflammatory states and OS cause a reduction in the bioavailability of nitric oxide (NO), a compound with antithrombotic, anti-inflammatory, and vasorelaxation action [18,19]. In particular, a NO reduction induced by the accumulation of NO synthetase endogenous inhibitors through the nuclear factor kappa-light-chain-enhancer of activated B cells (NF-kB) pathway was observed in CKD patients [20]. Furthermore, increased OS provokes the glycation of proteins, carbohydrates, and lipids and leads to the formation of advanced glycation end products (AGEs), which appear to have a direct toxic effect on the myocardium [21]. AGEs also increase the ED phenomenon by inhibiting the enzyme dimethylarginine dimethylaminohydrolase (DDAH), which degrades the ADMA, an endogenous inhibitor of NO production via reduction of the activity of endothelial NO synthetase [22,23,24].

Extra virgin olive oil (EVOO) plays a pivotal role among the functional foods that seem to reduce the risk of CV morbidity and mortality. In fact, the European Prospective Investigation (EPIC)-Spain study [25], the PREvención con DIeta MEDditerránea (PREDIMED) study [26], and the EUROLIVE study [27] have all highlighted the important cardioprotective action of EVOO. This beneficial effect seems to be related to the presence of minor phenolic compounds (MPCs), such as oleocanthal, oleacin, tyrosol and hydroxytyrosol (HT), and monounsaturated fatty acids (MUFAs), in which EVOO is rich [28,29,30]. In fact, these compounds exert an anti-inflammatory, antioxidant, and antiatherogenic effect, reducing the global CV risk. 

To date, we have tested two EVOOs other than the one selected for this clinical study. In particular, we previously administered two EVOOs to CKD patients—the first was characterized by a medium (>400 ppm) MPC content and the second one was characterized by a high (>700 ppm) MPC content—in order to assess EVOO’s potential beneficial effects on quality of life and on CKD-related comorbidities [31,32]. Moreover, in the study conducted by Noce et al. [32], we assessed whether the beneficial effects were maintained over time, namely after a two-month washout period. At this regard, we demonstrated that only the consumption of EVOO with a high MPC content resulted in the positive effects being maintained over time. 

In the light of our previous results, in this study, we evaluated the possible cardioprotective action of an EVOO with a very high (>900 ppm) MPC content in CKD patients under conservative therapy. This EVOO has never been tested before in in vivo study. Furthermore, in this study, we examined several parameters that were not investigated in our previous studies, namely atherogenic indices, carotid intima-media thickness (CIMT), and some inflammatory biomarkers (namely TNF-α, platelet-to-lymphocyte ratio (PLR), neutrophil-to-lymphocyte ratio (NLR), and lymphocyte-to-monocyte ratio (LMR)). In addition, in this study, we conducted a preliminary in vitro test on an extract, rich in oleocanthal, obtained from the selected EVOO in order to evaluate the effective anti-inflammatory activity of our EVOO.

## 2. Materials and Methods

### 2.1. Selection and Characterization of the Organic EVOO

For this study, we selected an organic EVOO from Ophena (Abruzzo), a town in central Italy, with a very high content of active MPCs [31]. This EVOO is produced from a mix of two cultivars of *Olea europaea* L., namely Leccino and Intosso, which are widespread in the Abruzzo region. 

This EVOO was produced using a biphasic and controlled pressing technology in order to preserve and maintain the very high content of active MPCs. The selected EVOO was analyzed using high-performance liquid chromatography coupled with diode array detector mass spectrometry (HPLC-DAD-MS) (Agilent Technologies, Palo Alto, CA, USA) to evaluate the contents of single active molecules such as HT, tyrosol, elenolic acid and its derivatives, oleacin, oleocanthal, oleuropein aglycone, secoiridoid derivatives, and lignans. The analyses were performed according to the protocol described by Romani et al. [31].

EVOO product analysis was carried out using the Oxitester system (CDR srl Florence, Italy) for parameters such as acidity (expressed as % of oleic acid), peroxides (expressed as meq O_2_/kg), and total polyphenols (expressed in mg of tyrosol) [31].

#### 2.1.1. Olea Extract

The used EVOO for the extraction had a total MPC content of 1145.95 mg/kg. The extract tested in this study was obtained using a liquid/liquid multiple extraction method starting with 10 L of EVOO. The selected EVOO blend was extracted with a 70% EtOH-H_2_O solution adjusted to pH 3.2 with formic acid in the dark for 30 min at room temperature in a mechanical orbital shaker. The solution was transferred to a separating funnel to remove the lipid portion with hexane. The liquid extract was dried under vacuum (Laborota 4000, Heidolph, Schwabach, Germany) and dissolved in 50 mL of 70% EtOH-H_2_O solution adjusted to pH 3.2 with formic acid. The obtained extract was mixed and dried again and then dissolved in 50 mL of H_2_O. The final redissolved extract was then degreased twice with hexane directly in the test tube and subjected to HPLC-DAD-MS analysis for qualitative and quantitative characterization.

#### 2.1.2. HPLC-DAD-MS Analysis of Olea Extract

The analyses for the qualitative and quantitative characterization of bioactive compounds of the Olea extract were achieved using an HP-1260 liquid chromatograph equipped with a DAD (Agilent-Technologies, Palo Alto, CA, USA). The HPLC system was interfaced with an Agilent MS system equipped with an ESI source (Agilent Corp, Santa Clara, CA, USA). The analyses were acquired in full-scan mode and the mass range was set to m/z 100–1500 in negative mode. The analytical columns and chromatographic methods used were described in a previous study of ours [31]. Polyphenols found in the extract were identified by comparing retention times and UV/Vis spectra with those of the authentic standards. Each compound was quantified at the selected wavelength (240, 280, 330, 350 nm) using a five-point regression curve and applying the correction of the molecular weights [31]. 

### 2.2. In Vitro Study

Murine macrophage RAW 264.7 cell line was purchased from the American Type Culture Collection (ATCC, Manassas, VA, USA). Cells were cultured in Dulbecco’s modified Eagle’s medium/high glucose (DMEM 4500, EuroClone, Milan, Italy) supplemented with 10% fetal bovine serum (FBS, EuroClone) and maintained at 37 °C in a humidified atmosphere containing 90% air and 10% CO_2_. Cells were harvested from subconfluent cultures via incubation with a trypsin-EDTA solution (EuroClone) and propagated every three days. Viability of the cells was determined by trypan blue exclusion test. Cultures were periodically monitored for Mycoplasma contamination using Chen’s fluorochrome test, as described in a previous study of ours [33]. 

#### 2.2.1. MTT Assay

Cell viability was assessed using a 3-(4,5-Dimethylthiazol-2-yl)-2,5-diphenyltetrazolium bromide (MTT) assay (Sigma Aldrich, Milan, Italy). Cells were placed into 96-well plates in complete medium in the absence and in the presence of different concentrations of oleocanthal standard (oleo std) or Olea extract that was rich in oleocanthal fraction (oleo fr) for 72 h. Next, MTT reagent was added to the medium and incubated at 37 °C. After 2 h, the blue MTT–formazan product was solubilized with dimethyl sulfoxide (DMSO, Sigma Aldrich) and its absorbance was read at 595 nm using a microplate reader (Bio-Rad, Milan, Italy), as previously described [34].

#### 2.2.2. Nitrite Assay

NO metabolite concentration was measured in the culture medium of RAW cells, using the Griess reaction, which is specific for nitrite dosage. First, 100 µL of cell culture medium from RAW264.7 cells was mixed with an equal volume of Griess reagent (1% sulfanilamide, 0.1% N-1-naphthalenediamine dihydrochloride, and 2.5% H_3_PO_4_) and transferred to 96-well plates. Plates were incubated at room temperature for 10 min. The absorbance was then measured at 540 nm in a microplate reader (BioTek, Winooski, VT, USA). The amount of nitrite in the media was calculated from the sodium nitrite (NaNO_2_) standard curve. Results were normalized to protein concentration. For RAW264.7 cells, nitrite production was expressed with reference to lipopolysaccharide (LPS; Sigma) as 100% [33].

#### 2.2.3. Western Blotting Analysis

Cells were lysed and separated using electrophoresis as previously described in the literature [35]. The primary antibodies were as follows: rabbit anti-inducible NO syntethase (iNOS) (1:1000, Cell Signaling Technology, Danvers, MA, USA) and rabbit anti-cyclooxigenase (COX)-2 (1:1000, Cell Signaling Technology, Danvers, MA, USA). The membrane was washed in T-PBS buffer, incubated for 1 h at room temperature with goat anti-rabbit IgG Alexa Fluor 750 antibody or with goat anti-mouse IgG Alexa Fluor 680 antibody (Invitrogen, Monza, Italy), and then visualized using an Odyssey Infrared Imaging System (LI-COR^®^ Bioscience, Lincoln, NE, USA). Mouse anti-alpha tubulin monoclonal antibody (1:1000, Cell Signaling Technology) was used to confirm that equal amounts of protein were loaded into each lane.

### 2.3. In Vivo Study

The aim of in vivo study was to evaluate the potential beneficial effects of an organic EVOO with a very high content of MPCs (>900 ppm) in CKD patients under conservative therapy.

#### 2.3.1. Sample Size

In this study, we enrolled 40 CKD patients under conservative therapy distributed according to a Gauss curve of known mean and unknown variance. Therefore, the aforementioned sample size of 40 patients derived from the following probabilistic considerations:

- The null hypothesis was rejected if the mean of the population from which the sample was extracted differed from the sample mean by an amount, expressed in absolute value, equal to or greater than 46.2% of the standard deviation;
- For the hypotheses’ (null and alternative) verification, the one-tailed Gauss z test with α = 0.05 (first type error) and β = 0.10 (second type error) was adopted in order to give a test power equal to 90%.

#### 2.3.2. Enrolled Patients

Consequently, 40 CKD patients under conservative therapy, stage I-IV (according to kidney disease: improving global outcomes—KDIGO guidelines [36]) were enrolled at the Hypertension and Nephrology Unit of Policlinico Tor Vergata (PTV), Rome (Italy). The inclusion criteria of study population were as follows: acceptance and signature of informed consent, age between 18 and 80 years, both sexes, and CKD stage I-IV. The exclusion criteria were as follows: the presence of either solid or hematological malignancies in the active phase, or of inflammatory and/or infectious pathologies.

**Figure 1:**
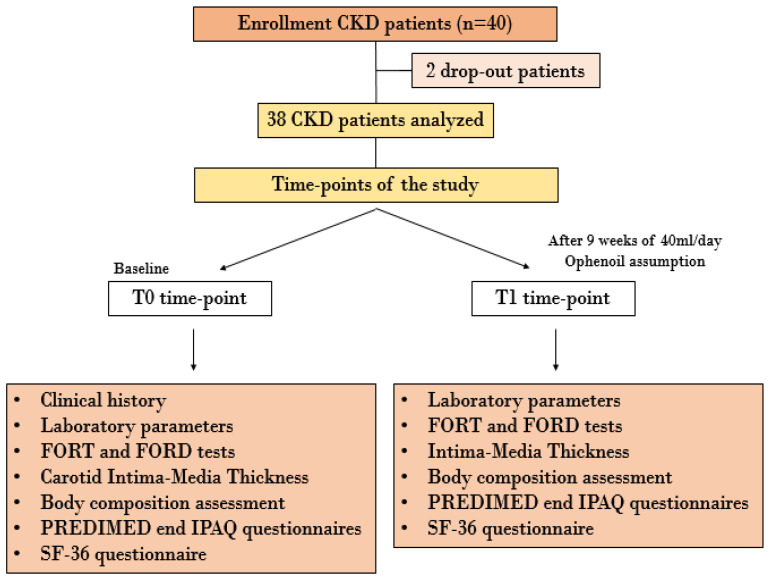
Flowchart of in vivo study. Abbreviations: CKD, chronic kidney disease; FORD, Free Oxygen Radical Defense; FORT, Free Oxygen Radical Test; IPAQ, International Physical Activity Questionnaires; PREDIMED, Preventiòn con dieta Mediterrànea; SF-36, Short Form Health Survey-36.

At the time of enrolment, all patients were instructed to consume 40 mL/day of the EVOO selected for clinical experimentation. The EVOO was to be consumed raw in the patients’ culinary preparations [37]. The study lasted 9 weeks. At baseline (T0) and after 9 weeks (T1), all the enrolled patients underwent blood and urinary analysis, body composition assessment via bioelectrical impedance analysis (BIA), Doppler ultrasound imaging of epiaortic vessels, monitoring of systolic and diastolic blood pressure, and evaluation of style and quality of life through specific questionnaires described below. The study protocol was declared to be compliant with the Helsinki Declaration by the PTV Independent Ethics Committee (protocol number 36/20). The flowchart of the study is represented in Figure 1.

#### 2.3.3. Blood and Urinary Analysis

At baseline (T0) and after 9 weeks (T1), all patients were assessed via blood and urinary analysis. In particular, glycemia, uricemia, intact parathyroid hormone, serum albumin, renal function parameters (like creatininemia, azotemia, albuminuria), electrolytes (like potassium, calcium, phosphorus, sodium), and lipid profile (like total cholesterol (TC), low-density lipoprotein cholesterol (LDL-C), high-density lipoprotein cholesterol (HDL-C), and triglycerides) were detected. Moreover, the patients underwent evaluation of inflammatory status via C-reactive protein (CRP), erythrocyte sedimentation rate (ESR), IL-6, TNF-α, PLR, NLR, and LMR and evaluation of OS biomarkers (like free oxygen radical defense (FORD) and free oxygen radical test (FORT)). In particular, the last ones allowed us to evaluate the antioxidant defenses and OS of the patients by means of capillary sampling, with a CR4000 instrument used for analysis [38].

#### 2.3.4. Atherogenic Indices

At both time-points of the study, we calculated the atherogenic indices. In particular, we calculated TC/HDL-C, LDL-C/HDL-C, and log(triglycerides/HDL-C) indices. These indices can provide additional information regarding CV risk, as they are closely related to the patient lipid profile [39].

#### 2.3.5. Body Composition Assessment

At T0 and T1, all patients underwent an anthropometric parameter assessment. Body weight was measured to the nearest 0.01 Kg using a balance (Seca 711, Hamburg, Germany), and height was measured using a stadiometer to the nearest 0.1 cm (Seca 220, Hamburg, Germany). Subsequently, the body mass index (BMI) was calculated as weight divided by the height squared. 

Moreover, the study population underwent BIA to evaluate the body composition at T0 and at T1. The BIA parameters were determined at 50 KHz frequency, using a BIA 101 S instrument (Akern/RIL System, Florence, Italy). In particular, resistance, reactance, phase angle, fat mass (FM), fat-free mass (FFM), intracell water (ICW), extracell water (ECW), total body water (TBW), body cellular mass (BCM), body cellular mass index (BCMI), and basal metabolic rate (BMR) were evaluated [40].

#### 2.3.6. Doppler Ultrasound of Epiaortic Vessels

All nephropathic patients underwent Doppler ultrasound to estimate CIMT. This examination was done using a MyLab70 VXG ultrasound device (Esaote, Genova, Italy) with a linear LA523 probe, at a 2–9 MHz frequency range. All examinations were conducted by the same operator (A.N.) to rule out operator-dependent bias. The CIMT was measured through a B-mode ultrasound examination at the level of the right common carotid artery. A longitudinal section of the right common carotid artery was obtained; three different CIMT measurements were performed, about 1 cm below the bifurcation, in the plaque-free area on the distal wall of the right common carotid artery, using a semiautomatic application. The CIMT was calculated as the average value of the three different measurements. This procedure was executed in accordance with the Mannheim protocol [41].

#### 2.3.7. Questionnaires

Three questionnaires were administered to all enrolled patients, evaluating both their lifestyle (PREDIMED and International Physical Activity Questionnaires-IPAQ) and their quality of life (Short Form 36 Health Survey-SF-36), at T0 and at T1. 

The lifestyle questionnaires were administered in order to exclude any bias due to lifestyle changes during the study period. In particular, the PREDIMED questionnaire monitors eating habits and adherence to the Mediterranean diet, while the IPAQ monitors the degree of physical activity of the patient [26,42]. 

The SF-36 questionnaire evaluates the quality of life as perceived by the patient through 36 questions regarding nine areas: general health, health change, physical functioning, pain, social functioning, emotional well-being, energy/fatigue, role limitation due to physical health, and role limitation due to emotional problems.

#### 2.3.8. Statistical Analysis

All data were entered into an Excel spreadsheet (Microsoft, Redmond, Washington, DC, USA) and the analyses were done using the Windows Social Science Statistics Package, version 25.0 (IBM_SPSS, Chicago, IL, USA). 

The descriptive analysis results are represented as mean ± standard deviation (SD) for the parameters with normal distributions (after confirmation with the histograms and the Kolgomorov–Smirnov test), while for the non-normal variables, the median and the interval (minimum:maximum) are used. 

The comparisons between the normal variables were done with one-way ANOVA, while the comparisons between the two time-points of the study (T0 versus T1) were performed using paired T-tests for normal variables, while Mann–Whitney tests were used for non-normal variables. Regarding occurrences (percentages), the chi-square test, possibly corrected by Fisher’s exact test, was applied. A *p* value < 0.05 was considered statistically significant. 

## 3. Results

### 3.1. Qualitative and Quantitative Characterization of the Organic EVOO

The HPLC-DAD-MS characterization of the EVOO selected for this study showed a very high content of MPCs, equal to 1145.98 mg/L. By evaluating the single molecules, we confirmed that the EVOO met the criteria required in order to claim CV protection according to the European Food Safety Authority (EFSA), as reported in Commission Regulation n. 432/2012 [43,44]. This regulation establishes that a daily intake of 20 g EVOO containing 5 mg of HT and its derivatives protects against blood lipid oxidation. In particular, in the EVOO selected for this study, the content of HT and its derivatives reached 778.43 mg/L. Moreover, in the EVOO administered in this study, we detected the contents of oleacin (315.46 mg/L) and oleocanthal (197.84 mg/L), molecules characterized by important anti-inflammatory activities [45,46]. 

Table 1 shows the chemical characterization of the single compounds identified in the EVOO sample.

Analyses relating to qualitative parameters of EVOO were carried out using the Oxitester system. We obtained low peroxide values (4.98 meqO_2_/kg) and acidity equal to 0.17%, indicating a high quality of the selected product (Table 2).

All results are expressed as average of three determinations. The standard error was <3% for acidity, <5% for peroxides, and <10% for polyphenols.

#### Oleocanthal Fraction Characterization

The HPLC-DAD-MS characterization analyses of the Olea extract showed high contents of oleacin (411.73 mg/g) and oleocanthal (331.73 mg/g) (Table 3). As we previously reported, for the analyzed EVOO sample, the high presence of these molecules permitted us to hypothesize a possible anti-inflammatory action of this extract. In fact, this action is added to the massive antioxidant properties already ascribed to these molecules and to HT, tyrosol, elenolic acid, oleuropein aglycone, ligstriside, and secoiridoidic derivatives [28,47,48].

### 3.2. In Vitro Study

To evaluate whether the oleo fr extracted from EVOO was more effective in terms of anti-inflammatory properties than the oleocanthal standard (oleo std), we compared their potential toxicities. In this way, equimolar doses of both oleo std and fr were administered to RAW 264.7 cells for 72 h and cell viability was determined via MTT assay. 

**Figure 2:**
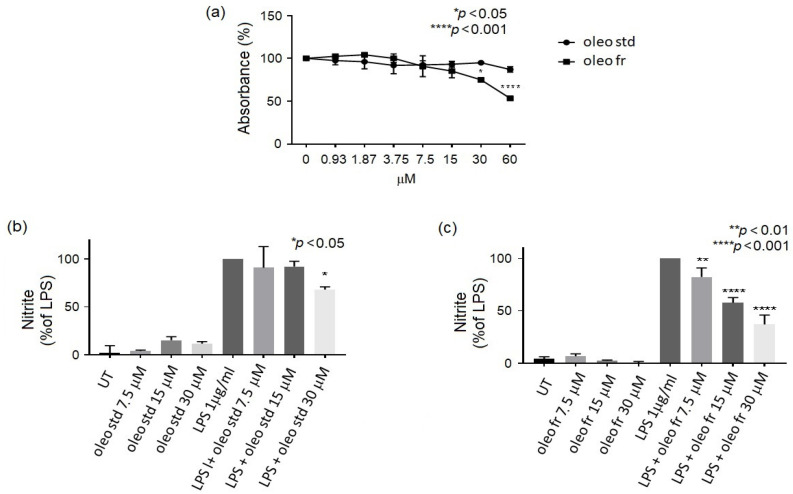
Cell viability assessed by MTT assay. (**a**) Cell viability assessed by MTT assay after 72 h of exposure to different doses of oleocanthal fraction (oleo fr) or oleocanthal standard (oleo std). Significance is indicated with * and relates to the untreated control (UT). Statistics were determined using two-way ANOVA and Sidak’s multiple-comparisons test; (**b**) Nitrite production by RAW 264.7 treated for 24 h with 1 μg/mL LPS and oleo std at different doses. Significance is indicated with * and relates to LPS as 100% of nitrite production. Statistics were determined using ordinary one-way ANOVA and Dunnett’s multiple-comparisons test; (**c**) Nitrite production by RAW 264.7 treated for 24 h with 1 μg/mL LPS and oleol fr at different doses. Significance is indicated with * and relates to LPS as 100% of nitrite production. Statistics were determined using ordinary one-way ANOVA and Dunnett’s multiple-comparisons test.

**Figure 3:**
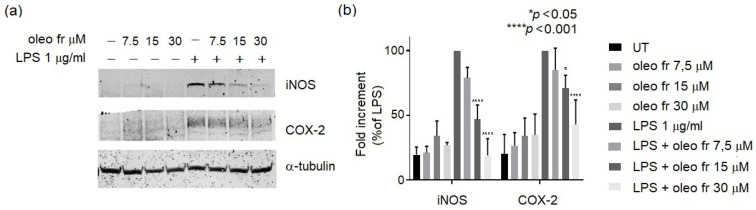
(**a**) Representative western blot panels of iNOS and COX-2 protein levels. Each band in the western blot was quantified via densitometric analysis and (**b**) the corresponding histogram was constructed by normalizing the density of each band to that of α-tubulin. The significance is indicated with * and relates to LPS. Statistical analyses were conducted using two-way ANOVA and Dunnett’s multiple-comparisons test.

Figure 2A shows that oleo fr was nontoxic up to a 15 μM dose, whereas oleo std did not affect cell viability even at high doses. To assess the anti-inflammatory property of oleo fr, we measured NO metabolite (specifically nitrite) production induced by 1 μg/mL of LPS (a marker of inflammation) in the same RAW 264.7culture medium, in either the presence or the absence of oleo fr. Oleo fr showed a higher capability to reduce NO production (Figure 2C) compared to oleo std (Figure 2B) and also compared to LPS, which was used as the positive control for nitrite production. In fact, it was already effective at a dose of 7.5 μM, compared to 30 μM of the oleo std. To study the anti-inflammatory capability of oleo fr in more depth, we also analyzed the expression levels of two enzymes directly implicated in the inflammatory response: iNOS and COX-2. Western blotting and densitometric analysis showed significant decreases of both iNOS and COX-2 expression, starting from a concentration of 15 μM (Figure 3).

These results suggest a greater effectiveness of the fraction compared to the standard, probably due to the synergistic effect induced by all components present in the fraction.

### 3.3. In Vivo Study

The main epidemiological features of the study population (40 CKD patients under conservative therapy) are shown in Table 4. In particular, the mean age of the study population was 70.7 ± 10.6 years (CI: 49.5–80). The values of systolic and diastolic blood pressure monitored in the different time-points did not demonstrate any statistical significance.

Only 38 patients completed the clinical trial. We recorded two drop-outs, for reasons not related to the experiment.

Table 5 shows the results of the laboratory and urinary parameters assessed at T0 and at T1. At the end of the study (T1), we observed an improvement of renal function parameters, with a significant reduction of serum level of creatinine (*p* = 0.013), azotemia (*p* = 0.017), and albuminuria (*p* = 0.027). Moreover, we noticed an amelioration of lipid and purine metabolism, with a significant increase of HDL-C and a significant decrease of triglycerides (*p* = 0.002) and uric acid serum levels (*p* = 0.039). The results also showed an improvement in the nutritional status of the patients after EVOO consumption, with a significant increase in serum albumin levels (*p* = 0.001). 

As for the OS and the inflammation parameters, after 9 weeks of EVOO consumption, we observed significant reductions of FORT level (*p* = 0.013) and of CRP (*p* = 0.046), ESR (0.0006), TNF-α (*p* = 0.0001), and IL-6 (*p* = 0.019) serum levels, as reported in Table 6. In contrast, the patients’ antioxidant defenses seemed to remain stable.

Table 7 expresses the data regarding atherogenic indices. At the end of the study, we observed significant reductions of TC/HDL-C (*p* = 0.0171), LDL-C/HDL-C (*p* = 0.0098), and log(triglycerides/HDL-C) (*p* = 0.0067).

After 9 weeks of EVOO consumption, we also observed an amelioration in the other inflammatory parameters, namely the PLR (*p* = 0.0003) and LMR (*p* = 0.0406), and in the absolute number of lymphocytes (*p* = 0.0007), as reported in Table 8. 

The Doppler ultrasound imaging showed a significant reduction of CIMT (*p* = 0.017), as summarized in Table 9.

Finally, the body composition assessment by BIA highlighted a significant increase of reactance (0.007) and of BCM % (*p* = 0.025), as reported in Table 10. 

**Figure 4:**
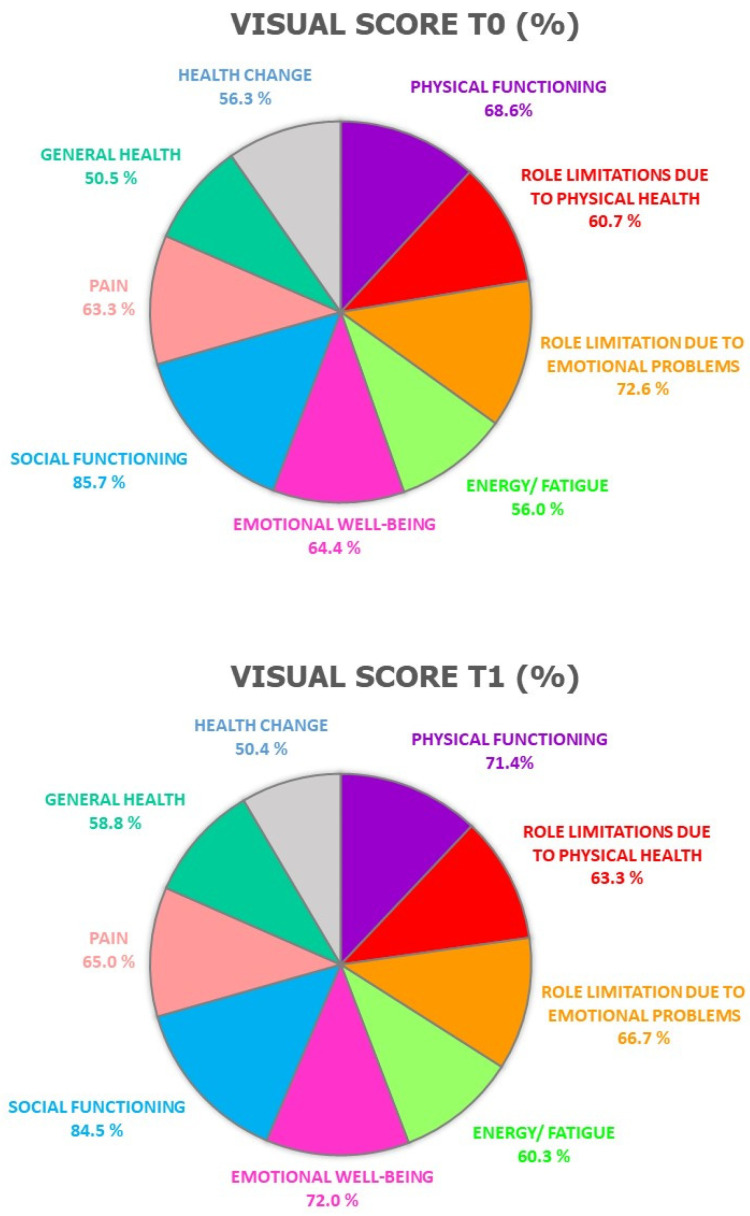
Visual scores, expressed in % for each sphere, of the SF-36 questionnaire administered at baseline and after 9 weeks of study.

The lifestyle questionnaires PREDIMED and IPAQ did not show statistically significant differences between the two observation time-points, highlighting that the results obtained may be attributable to EVOO consumption. Moreover, at baseline (T0) and after 9 weeks (T1) of in vivo study, the SF-36 questionnaire was administered to assess patients’ perception of their quality of life. At T1, we observed an improvement in the general health and emotional well-being spheres (Figure 4).

## 4. Discussion

In our study population, we observed a significant improvement of lipid metabolism after 9 weeks of treatment with an EVOO with a very high MPC content. In particular, we observed a significant increase of HDL-C and a reduction of triglycerides. These results could be partly related to the high content of HT. In fact, in a previous animal study, Jemai et al. [49] demonstrated a lipid-lowering action of HT and its derivatives, related to their antioxidant effects which counteract the lipid peroxidation. Moreover, Cao and co-authors [50] showed that HT supplementation, at a dose of 50 mg/kg/day for 17 weeks in obese mice, induced a decrease of sterol regulatory element-binding protein 1 (SREBP-1c) levels, which is a cholesterol-sensitive transcription factor that stimulates the activity of hydroxy-methyl-glutaryl-coenzyme A (HMG-CoA) reductase. In subsequent human studies, conducted on metabolic syndrome patients treated with oral food supplements with a high HT content [51,52], the hypolipemic effect of HT was confirmed. 

Interestingly, in our study population, we also observed an amelioration of atherogenic indices. In detail, we detected significant decreases of TC/HDL-C, LDL-C/HDL-C, and log (triglycerides/HDL-C). These data are in accordance with the results obtained in the EUROLIVE Study [27], which reported an amelioration of fatty acid profile in LDL-C, probably correlated to a decrease in lipid oxidative injury. The observed reduction of TC/HDL-C is very important as it highlights the possible cardioprotective action of EVOO with a very high MPC content in nephropathic patients. In fact, this ratio represents a milestone biomarker in lipid atherogenesis, because it is linked to the balance between the passage of cholesterol in and out of the arterial intima [53]. Moreover, the Framingham Offspring Study showed that TC/HDL-C and LDL-C/HDL-C are the most important biomarkers for CV events, among all lipid parameters [54].

In the light of the CV protection induced by the EVOO administered to our study population, it was of utmost importance to observe not only an amelioration of lipid profile, but also a reduction of the chronic inflammatory state typical of CKD patients. In fact, atherosclerotic CV disease cannot be considered only a pathological condition related to the cholesterol accumulation, but is characterized by a more complex pathogenic mechanism and is frequently triggered by a chronic inflammatory status [55]. Inflammatory cell infiltrates have been isolated in atherosclerotic plaque [56], corroborating this hypothesis. Moreover, in the Physicians’ Health Study [57] and Women’s Health Study [58], a direct association was shown between pro-inflammatory cytokine (such as IL-6 and IL-1β) levels and atherosclerotic CV disease. In our study, we highlighted significant reductions of CRP, ESR, TNF-α, and IL-6 after 9 weeks of EVOO administration, confirming the cardioprotective action of EVOO with a very high MPC content in CKD patients. This anti-inflammatory effect was related to the high content of oleocanthal in the EVOO selected. In fact, several studies have pointed out the anti-inflammatory action induced by oleocanthal, as it is an unselective inhibitor of COX-1 and -2 [46,59]. This anti-inflammatory action is also supported by our in vitro data, as discussed above.

In addition, the inflammatory status improvement seemed to positively influence the degree of ED related to CKD. In fact, in the initial stages of atherosclerotic disease, pro-inflammatory cytokines (like IL-6 and TNF-α, which are significantly reduced at T1 in our study) play a key role. These molecules, on one hand, induce the migration of leukocytes into the subendothelial space and, on the other hand, alter the gene expression of the adhesion molecules. The latter, in turn, favor the adhesion, chemotaxis, and subendothelial migration of the same leukocytes [60]. In detail, the physiopathological process that induces atherosclerotic plaque formation is mediated by the accumulation of monocytes in the subendothelial space [61]. We consider the reduction of CIMT observed at the end of our study to support this hypothesis. In detail, upon analyzing the data obtained from the ultrasound examination of the epiaortic vessels, we detected a CIMT improvement in patients presenting a slight increase of CIMT, but not in those who had already developed a carotid atherosclerotic plaque. In this regard, in the carotid atherosclerosis progression study (CAPS), the authors observed a direct correlation between serum CRP levels and an increase in CIMT [62]; at the T1 time-point, we registered a significant decrease of CRP levels, as previously described. In addition to traditional inflammatory biomarkers such as ESR and CRP, we also calculated, the following ratios in order to monitor the inflammatory state: NLR, PLR, and LMR. The latter parameters, which are easy and quite cheap to monitor, were significantly decreased at the end of the study. Furthermore, they are associated with the inflammatory state of the subject and they seem to be correlated to the CV risk. In particular, NLR appears to be a reliable predictor of cardiac arrhythmia and it represents a short- and long-term biomarker of CV disease [63]. On the other hand, PLR seems to be an integrated parameter able to monitor both thrombotic and inflammatory pathways [64]. Historically, it has been used as inflammatory biomarker in cancer patients [65,66], and a possible role has only recently identified as a predictor for CV events [67]. Finally, in a human study conducted on 199 patients that underwent coronary angiography, LMR was associated directly and independently with the severity of coronary atherosclerosis [68]. Therefore, the reduction of these three ratios observed in our study allows further speculation regarding the strong CV protection induced by EVOO with a very high MPC content. A further possible explanation of the CIMT improvement relies on the reduction of LDL-C/HDL-C observed. In fact, a meta-analysis demonstrated that this ratio is related to CIMT. Moreover, low HDL-C concentrations seem to be inversely related to CIMT [69].

In this paper, we confirmed the positive impact of EVOO on slowing CKD progression, as already demonstrated in our previous studies [16,32]. In fact, we observed at T1 a significant reduction of creatinine and uric acid, and an increase of estimated-GFR. Moreover, at the end of the study, we noticed a reduction of microalbuminuria, which on one hand represents a biomarker of renal dysfunction and CKD progression [29,31,70], and on the other hand is related to the CV risk [71]. Therefore, its significant reduction confirms both the cardioprotective and nephroprotective action of EVOO. 

We also obtained an additional result, namely a reduction of OS as monitored by FORT. This interesting observation supports the cardioprotective action induced by EVOO and it is probably related to its high content of oleocanthal. This molecule also exerts an antioxidant action, reducing the activity of nicotamide adenine dinucleotide phosphate oxidase and, at the same time, ROS production [72].

Finally, we showed an improvement of the nutritional status of patients, related to the significant enhancement of albuminemia observed at the end of the study. In fact, hypoalbuminemia is frequently present in CKD patients under conservative therapy, and it is correlated with both a low caloric intake and a chronic inflammatory status. In this regard, EVOO consumption at a dose of 40 mL/day is able to counteract the inflammation and, at the same time, provide the correct caloric intake [73].

Moreover, regarding body composition assessment, we observed a significant increase of BCM% after our intervention. This parameter is not only directly related to albumin, as previous demonstrated in the literature, but it is a useful tool to evaluate inflammation in CKD and elderly patients [74]. 

As for the SF-36 questionnaire, this test allowed us to underline an enhancement of quality of life as perceived by patients, likely related to their enhanced health status.

## 5. Conclusions

We would like to stress that this study highlighted the cardioprotective action of EVOO in nephropathic patients for the first time. This positive health effect could be due to the reduction of the chronic inflammatory state produced by the oleocanthal and to the improvement of lipid metabolism induced by HT and oleuropein.

Previous clinical trials (such as PREDIMED [26], EPIC [25], and EUROLIVE [27]) have shown this beneficial effect only in other CV high-risk populations, but not in CKD patients.

In order to reduce CV mortality in CKD, the traditional nutritional treatment, planned according to CKD stage, should be combined with functional foods. Among these, according also to EFSA’s health claim [43,44], EVOO with a high content of MPCs seems to be one of the most important functional foods exerting cardioprotective action.

## Figures and Tables

**Table 1:** HPLC-DA-MS analysis of Ophena EVOO.

Compounds	EVOO mg/L
Hydroxytyrosol	3.10 ± 0.09
Tyrosol	1.02 ± 0.03
Elenolic Acid Derivatives	9.31 ± 0.28
Elenolic acid	150.06 ± 4.50
Oleacin (10-hydroxy-oleocanthal)	315.46 ± 9.46
Oleocanthal	197.84 ± 5.04
Secoiridoid derivatives	96.43 ± 2.89
Lignans	208.17 ± 6.25
Oleuropein aglycone	164.58 ± 4.94
Total MPCs	1145.97 ± 34.38
MPCs excluding elenolic acid and derivatives	986.60 ± 29.60

Abbreviation: MPCs, minor phenolic compounds.

**Table 2:** Acidity, peroxides, and polyphenols of EVOO.

EVOO	
Acidity (% acid oleic)	0.17
Peroxides (meqO_2_/kg)	4.98
Polyphenols (mg tyrosol/kg)	890

**Table 3:** HPLC-DAD-MS characterization of Olea extract.

Olea Extract	mg/g
Hydroxytyrosol	7.93 ± 0.24
Tyrosol	7.12 ± 0.22
Elenolic acid	89.19 ± 2.68
Oleacin (10-hydroxy-oleocanthal)	411.73 ± 12.35
Oleocanthal	331.73 ± 9.95
Oleuropein aglycone	75.87 ± 2.27
Ligstroside	4.93 ± 0.15
Secoiridoid derivatives	59.03 ± 1.77
Total	987.53 ± 29.63

**Table 4:** Epidemiological features of study population.

N°	40
Age (years)	70.7 ± 10.6
Sex (M/F)	24/16
BMI (kg/m^2^)	27.6 ± 5.2

Data are expressed as mean ± standard deviation. Abbreviation: BMI, body mass index.

**Table 5:** Laboratory and urinary parameters assessed at baseline and after 9 weeks of study.

	T0	T1	*p*-Value
Creatinine (mg/dL)	2.17 ± 0.17	2.08 ± 0.16	0.013
e-GFR (mL/min/1.73 m^2^)	33.73 ± 2.81	35.35 ± 2.96	0.040
Albuminuria (mg/dL)	223.43 ± 60.95	120.88 ± 32.35	0.027
Albumin (g/dL)	4.22 ± 0.26	4.38 ± 0.31	0.001
Azotaemia (mg/dL)	79.03 ± 5.86	69.82 ± 4.69	0.017
Sodium (mEq/L)	141.24 ± 2.24	136.62 ± 2.34	n.s.
Potassium (mEq/L)	4.59 ± 0.64	4.69 ± 0.37	n.s.
Calcium (mg/dL)	9.51 ± 0.53	9.46 ± 0.50	n.s.
Phosphorus (mg/dL)	3.66 ± 0.63	3.67 ± 0.61	n.s.
TC (mg/dL)	176.20 ± 40.51	175.91 ± 38.64	n.s.
HDL-C (mg/dL)	45.26 ± 1.81	49.02 ± 2.29	0.004
LDL-C (mg/dL)	105.74 ± 37.22	103.71 ± 32.33	n.s.
Triglycerides (mg/dL)	124.45 ± 9.71	111.03 ± 8.27	0.002
PTHI (mg/dL)	90.15 ± 28.80	75.63 ± 11.77	n.s.
Glycaemia (mg/dL)	95.39 ± 27.01	100.97 ± 30.17	n.s.
Uric acid (mg/dL)	6.34 ± 0.31	5.68 ± 0.29	0.039

Data are expressed as mean ± standard deviation; Abbreviations: E-GFR, Estimated Glomerular Filtration Rate; TC, Total Cholesterol; HDL-C, High-Density Lipoprotein Cholesterol; LDL-C, Low-Density Lipoprotein Cholesterol; n.s., Not Significant; PTHI, Parathyroid Hormone Intact.

**Table 6:** Oxidative stress and inflammatory parameters assessed at baseline and after 9 weeks of study.

	T0	T1	*p*-Value
FORT (U)	397.60 ± 27.74	303.17 ± 25.65	0.013
FORD (mmol/L Trolox)	1.37 ± 0.49	1.51 ± 0.50	n.s.
CRP (mg/L)	5.39 ± 1.22	3.13 ± 0.82	0.046
ESR (mm/h)	46.43 ± 4.70	39.22 ± 3.96	0.0006
TNF-α (pg/mL)	89.70 ± 66.53	36.65 ± 32.83	0.0001
IL-6 (pg/mL)	71.22 ± 23.81	10.13 ± 2.50	0.019

Data are expressed as mean ± standard deviation. *t*-test was applied for paired data. Values of *p* ≤ 0.05 are considered statistically significant. Abbreviations: CRP, C-Reactive Protein; ESR, Erythrocyte Sedimentation Rate; FORD, Free Oxygen Radical Defense; FORT, Free Oxygen Radical Test; IL, Interleukin; n.s., Not Significant; TNF, Tumor Necrosis Factor.

**Table 7:** Atherogenic indices.

	T0	T1	*p*-Value
TC/HDL-C	3.90 (2.62–6.58)	3.42 (2.31–6.57)	0.0171
LDL-C/HDL-C	2.27 (1.20–5.47)	1.91 (1.20–4.15)	0.0098
log(triglycerides/HDL-C)	0.05 (0.03–0.11)	0.04 (0.02–0.09)	0.0067

Data are expressed as a median and the minimum-maximum range; Abbreviations: TC, Total Cholesterol; HDL-C, High-Density Lipoprotein Cholesterol; LDL-C, Low-Density Lipoprotein Cholesterol; n.s., Not Significant.

**Table 8:** Other inflammatory parameters.

	T0	T1	*p*-Value
Platelet-to-lymphocyte ratio	135.09 (51.08–337.84) ^a^	116.01 (55.08–227.00) ^a^	0.0003
Neutrophil-to-lymphocyte ratio	2.22 (0.75–7.00) ^a^	2.14 (0.86–7.45) ^a^	n.s.
Lymphocyte-to-monocyte ratio	3.75 (0.27–7.74) ^a^	3.50 (0.15–8.21) ^a^	0.0406
Lymphocytes (n/mm^3^)	2.03 ± 0.70 ^b^	1.78 ± 0.65 ^b^	0.0007

^a^ Data are expressed as a median and the minimum-maximum range; ^b^ Data are expressed as mean ± standard deviation. Abbreviation: n.s., Not Significant.

**Table 9:** CIMT values assessed at baseline and after 9 weeks of study.

	T0	T1	*p*-Value
Carotid Intima-Media Thickness (mm)	1.10 ± 0.44	1.02 ± 0.35	0.017

Data are expressed as mean ± standard deviation.

**Table 10:** Body composition parameters assessed at baseline and after 9 weeks of study.

	T0	T1	*p*-Value
Weight (kg)	74.1 ± 13.8	73.7 ± 13.7	n.s.
BMI (kg/m^2^)	27.76 ± 5.27	28.62 ± 4.50	n.s.
Resistance (ohm)	496.0 ± 80.3	493.6 ± 104.8	n.s.
Reactance (ohm)	40.78 ± 1.71	43.63 ± 1.80	0.007
Phase angle (°)	4.80 ± 0.86	4.90 ± 1.01	n.s.
TBW (%)	52.6 ± 9.1	72.4 ± 10.6	n.s.
ICW (%)	47.2 ± 5.3	49.1 ± 8.5	n.s.
ECW (%)	52.8 ± 5.3	50.9 ± 8.5	n.s.
FM (%)	28.6 ± 9.7	29.4 ± 10.6	n.s.
FFM (%)	69.6 ± 11.6	70.7 ± 9.4	n.s.
BCM (%)	44.6 ± 9.2	47.0 ± 6.4	0.025
BCMI	9.0 ± 1.7	9.1 ± 1.8	n.s.
BMR (Kcal)	1452.3 ± 154.7	1453.3 ± 157–5	n.s.

Data are expressed as mean ± standard deviation. Abbreviations: BCM, Body Cellular Mass; BCMI, Body Cellular Mass Index; BMI, Body Mass Index; BMR, Basal Metabolic Rate; ECW, Extra Cell Water, FM, Fat Mass; FFM, Fat Free Mass; ICW, Intra Cell Water; n.s., Not Significant; TBW, Total Body Water.

## Data Availability

Data supporting reported results can be found upon request to the corresponding author.

## Abbreviation

ADMA	Asymmetric Dimethylarginine
AGEs	Advanced Glycation End Products
BCM	Body Cellular Mass
BCMI	Body Cellular Mass Index
BIA	Bioelectrical Impedance Analysis
BMI	Body Mass Index
BMR	Basal Metabolic Rate
CAPS	Carotid Atherosclerosis Progression Study
CIMT	Carotid Intima-Media Thickness
CKD	Chronic Kidney Disease
COX	Cyclooxygenase Enzymes
CRP	C-Reactive Protein
CV	Cardiovascular
DDAH	Enzyme Dimethylarginine Di-Methylaminohydrolase
ECW	Extra Cell Water
ED	Endothelial Dysfunction
EFSA	European Food Safety Authority
EPIC	European Prospective Investigation
ESR	Erythrocyte Sedimentation Rate
EVOO	Extra Virgin Olive Oil
FFM	Fat Free Mass
FM	Fat Mass
FORD	Free Oxygen Radical Defense
FORT	Free Oxygen Radical Test
SF-36	Short Form 36 Health Survey
GFR	Glomerular Filtration Rate
HDL-C	High-Density Lipoprotein Cholesterol
HMG-CoA	Hydroxy-methyl-glutaryl-coenzyme A
HPLC-DAD-MS	High-Performance Liquid Chromatography Coupled with Diode Array Detector-Mass Spectrometry
HT	Hydroxytyrosol
ICW	Intra Cell Water
IL	Interleukin
IPAQ	International Physical Activity Questionnaires
KDIGO	Kidney Disease: Improving Global Outcomes
LDL-C	Low-Density Lipoprotein Cholesterol
LMR	Lymphocyte-To-Monocyte Ratio
LPS	Lipopolysaccharide
MPCs	Minor Phenolic Compounds
MTT	3-(4,5-Dimethylthiazol-2-yl)-2,5-diphenyltetrazolium bromide
MUFAs	Monounsaturated Fatty Acids
NF-kB	Nuclear Factor Kappa-Light-Chain-Enhancer of Activated B Cells
NLR	Neutrophil-To-Lymphocyte Ratio
NO	Nitric Oxide
Oleo fr	Oleocanthal fraction
Oleo std	Oleocanthal standard
OS	Oxidative Stress
PLR	Platelet-To-Lymphocyte Ratio
PREDIMED	Prevención Con Dieta Mediterránea
PTV	Policlinico Tor Vergata
ROS	Reactive Oxygen Species
SD	Standard Deviation
TBW	Total Body Water
TC	Total Cholesterol
TNF	Tumor Necrosis Factor
